# Accelerated Remote Consultation Tele-POCUS in Cardiopulmonary Assessment (ARCTICA)

**DOI:** 10.24908/pocus.v5i2.14452

**Published:** 2020-11-18

**Authors:** Jeffrey Lam, Sherwin Wong, Nicholas Grubic, Salwa Nihal, Julia E Herr, Daniel J Belliveau, Stephen Gauthier, Steven J Montague, Amer M Johri

**Affiliations:** 1 Department of Medicine, Division of Cardiology, Queen's University Kingston, Ontario Canada

**Keywords:** Point-of-Care Ultrasound, remote learning, tele-POCUS

## Introduction

The ability of point-of-care ultrasound (POCUS) to provide rapid and accurate bedside assessment of both the heart and lungs allows it to be a powerful tool in the management of patients presenting with dyspnea. However, while ultrasound equipment is readily available even in remote healthcare settings in Canada, physicians lack effective training opportunities to develop expertise in this potentially life-saving skill. To answer this critical call to action, we have developed the Accelerated Remote Consultation Tele-POCUS in Cardiopulmonary Assessment (ARCTICA) program to innovate POCUS training for today’s physician leaders. This article outlines the background, research methods, and progress-to-date of ARCTICA.

In the context of the COVID-19 pandemic, POCUS has a beneficial role in risk stratification. Although lung injury is common in COVID-19 infection, myocardial injury is present in >25% of patients with critical disease 1. Given the cardiopulmonary nature of this infection, application of POCUS enhances the outcomes of patients in COVID-19 2. Specifically, rapid and serial bedside detection of pulmonary and cardiac abnormalities allows tailoring therapy for COVID-19 patients. It has been well demonstrated that COVID-19 patients with myocardial injury have worse prognoses than those without 3, 4, and there is mounting evidence that structural changes identified with cardiac ultrasound have adverse prognostic implications 5. POCUS is recommended as the first-line imaging tool in the assessment of COVID-19. The application of POCUS has now been shown to enhance outcomes in COVID-19 infection 6. 

To develop POCUS expertise, physicians must engage in deliberate practice 7, 8. A vital component of this learning process is the acquisition of recurrent individualized feedback. However, most currently available POCUS training programs for practicing physicians are limited to one-time or single-day workshops 9, which poses significant barriers to continued skill development. This problem highlights the need for longitudinal training opportunities that integrate high-quality feedback into a physician’s everyday practice. This need is even more pressing for healthcare providers in rural or underserved communities where POCUS expertise is scarce but would be of high yield due to imaging access challenges.

The use of real-time, remotely supervised ultrasound (Tele-POCUS) to guide physicians with basic ultrasound competence is a useful clinical tool in settings where on-site supervision is unavailable 10, 11. Tele-POCUS implementation may be particularly useful for Indigenous and remote Northern Canada care centres, where access to professional cardiopulmonary services is limited. Although telehealth services show early promise to enhance access to care in remote Northern Canadian and rural communities 12, 13, there remains a significant gap in access to diagnostic services, especially ultrasonography 14. To these ends, we have proposed developing an entirely virtual POCUS training program for physicians that utilizes e-learning modules and remotely supervised Tele-POCUS to provide real-time feedback.

## Objective

**Figure 1:**
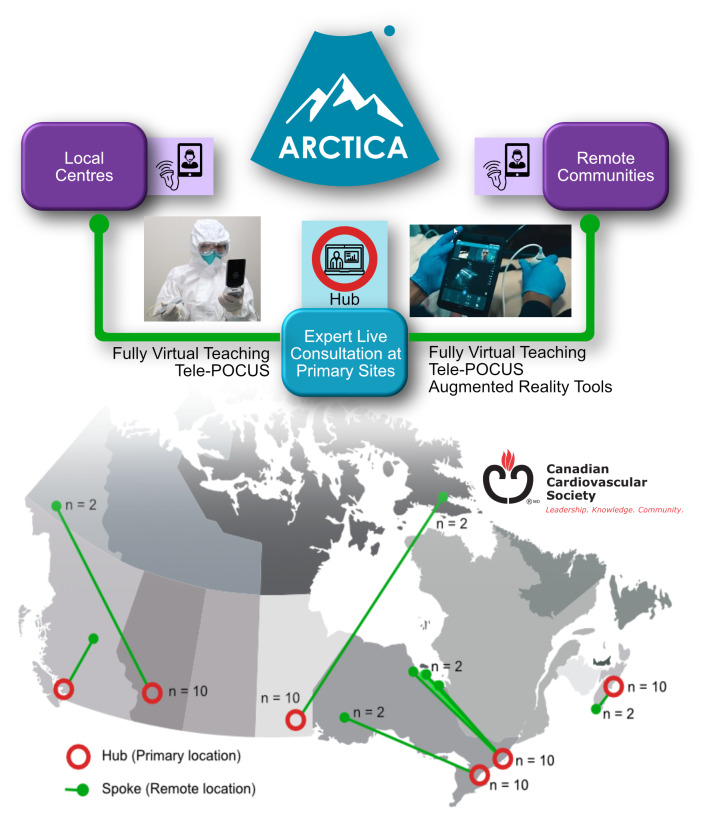
ARCTICA Hub-and-Spoke model of training and remote POCUS consultation.

The objective of ARCTICA is to evaluate the implementation of a Tele-POCUS training and workflow program provided to local non-expert users and geographically remote facilities for POCUS cardiopulmonary assessment of patients with cardiopulmonary complications. The program was initially designed to remotely train physicians in COVID-19 wards and offer them live expert support to conduct POCUS exams at the beside, thus reducing the need for patient transportation and decreasing the risk of exposure to additional personnel. However, the broader clinical application of ARCTICA as a support system for remote healthcare centres is apparent. The program has since been expanded to encompass cardiopulmonary POCUS assessments in dyspneic patients, thus providing valuable skills that would apply to a variety of cardiopulmonary complaints. The program will first be optimized at 6 academic centres, where lung and cardiac ultrasound experts will provide Tele-POCUS to general internal medicine (GIM) physicians caring for patients presenting with dyspnea. Each academic centre will then serve as an expert hub and will be assigned to a geographically remote or rural satellite spoke from which POCUS images will be streamed to obtain expert real-time consultation (Figure 1).

## Methods

Three POCUS modules (ultrasound basics, cardiac ultrasound, and lung ultrasound) have been designed to deliver e-learning to GIM physicians (https://cinqlab.com/research/research-studies/arctica/) 15. Physician participants are asked to review the modules and practice image acquisition on a volunteer at home over one week. Participants then have an additional one-hour virtual workshop run by an experienced POCUS Educator, where learners receive real-time supervision and feedback. Such sessions make use of Reacts, a software application created by Innovative Imaging Technologies Inc. (IIT) that allows the image acquired to be directly streamed to the educator’s device, which then directs probe angles, pressures, and window acquisition.

Following this first week of training, participants are assessed using a modified, objective structured assessment of ultrasound skills (OSAUS). The OSAUS is administered via a one-on-one virtual meeting by an Educator using Reacts. We developed the training standards for POCUS users defining the minimum number of scans and content required 16. The POCUS Educator will provide ongoing quality control and virtual classroom teaching at all sites.

**Figure 2:**
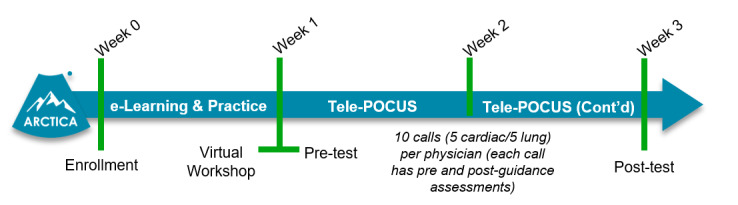
ARCTICA Participant Timeline. Upon enrolment, physicians will be provided with e-learning modules and instructed to practice independently (Week 0). After one week of self-directed learning, participants will partake in a live workshop led by expert imagers (Week 1). Following training (Week 1), physicians will be assessed through a POCUS pre-test (Week 1). Physicians will then participate in a two-week Tele-POCUS block (Weeks 1-2) making 10 calls to receive tele-mentored support from expert imagers. At the end of the study, physician POCUS skills will be tested again with a POCUS post-test (Week 3).

The Tele-POCUS service will be available continuously. For two weeks following training, physicians will initiate calls in clinical encounters where there is an indication for POCUS cardiopulmonary assessment. When a call is made to this service, the assigned ultrasound expert will receive real-time images to his/her phone and provide remote bedside guidance for image acquisitions and interpretation. During initial local workflow optimization, physicians are being surveyed after each Tele-POCUS encounter. The survey collects data on the ease of use, clinical integration, utility, and likelihood to continue use. Indications, outcomes, and feedback are also collected. Following the two-week Tele-POCUS block, physicians will be evaluated through a post-test OSAUS to assess clinical skill improvement. The ARTICA participant timeline is shown in Figure 2.

Following local optimization, the Tele-POCUS workflow is implemented in remote care facilities. POCUS devices (Lumify, Philips Healthcare) will be provided to each of the 6 academic hubs and remote spokes (Table 1). Implementation of the live-streaming Reacts application by non-expert physicians at all 12 sites will be evaluated. Ten physicians from each academic site and 2 from each remote site will be evaluated for a total of 72 physicians. At each location, entirely virtual POCUS training will be provided to physicians caring for the patient presenting with dyspnea. Designated research assistants within each site will coordinate with the central POCUS Educator to set up virtual hands-on scanning sessions and modules.

**Table 1:** Overview of academic expert hubs with the potential corresponding rural spoke site.

**Expert Hub**	**Potential Spoke Site**
Queen’s University	Weeneebayko Area Health Authority (WAHA)
University of Toronto	Sioux Lookout Meno Ya Win Health Centre (SLMHC)
Dalhousie University	Yarmouth Health Centre
University of Calgary	Whitehorse Health Centre
University of Manitoba	Qikiqtani General Hospital, Iqaluit
University of British Columbia	Prince George Health Centre

## Outcomes

**The primary outcome** will be the physician’s average improvement in the image acquisition proficiency scale. Each Tele-POCUS assessment call evaluates the operator’s skill in acquiring multiple views; to account for the multiple items (all scored from 1 to 5), each evaluation is given an average score of all views. As physician participants may have a varying number of tele-POCUS calls to experts (6-10), we will average the pre-guidance to post-guidance change in the tele-POCUS evaluation scores to each physician’s level before analysis. A one-sample t-test will be used to assess the physicians’ average difference in scores, i.e. the change in score of the average of each view per tele-POCUS call, averaged over calls. The average change in score will be reported with 95% confidence intervals. **The secondary outcome** will be the within-physician change in the 5 points skill test from before to after the tele-POCUS consultation period. The average improvement in the test score will be assessed using the Wilcoxon signed-rank test. 

## Progress to Date (Pilot Data)

Our team has received the Canadian Cardiovascular Society (CCS) COVID-19 Challenge for Canada Award, which has allowed us to assess feasibility at our primary hub site at Queen’s University. This permitted us to develop all learning modules, obtain Research Ethics Board (REB) clearance, purchase two Philips Lumify devices, and begin a pilot study recruiting GIM physicians. The learning modules have been specifically tailored to the knowledge base of GIM physicians and have been streamlined into three specific modules: Ultrasound Theory and Basics, Cardiac Point-of-Care ultrasound, and Pulmonary Point-of-Care ultrasound. The pertinent information related to the pathologies expected in patients presenting with dyspnea has been emphasized in each module, with additional details regarding COVID-19-specific safety measures given the current pandemic. The Kingston site has two Expert Physicians available to receive Tele-POCUS consultation calls, a POCUS Educator, a Research Coordinator to manage participant recruitment and data collection, and a team of researchers and medical trainees assisting with daily operations of the study. The Kingston site anticipates the enrollment and study completion of eight participants by the end of 2020, and is actively working with the Weeneebayko Area Health Authority spoke site to begin the remote training program.

## Conclusions

ARCTICA will provide lasting infrastructure, build expert relationships, and provide a mechanism for ongoing quality control in the education of a standard POCUS curriculum for practicing physicians. The Hub and Spoke model brings state-of-the-art technology directly into a new user’s hands and to those in underserviced and Northern communities. We will be providing ultrasound probes and tablets for caregivers with an expected shelf life of 6-8 years; the portable nature of the devices allows for scaling up to a broader geographic area. It is critical to note that we are not providing technology in isolation resulting in the loss of benefit to the community when the project ends. We provide lasting skills to caregivers that remain in the community so that they will be prepared to use POCUS to guide clinical decisions and for future pandemics after the project ends. They will be equipped to teach others within their healthcare communities, ensuring there is an inherent wellspring of knowledgeable users. By linking care providers in spoke communities with experts in hubs, we formalize professional links and build lasting rapport for these previously underserved regions.

## Conflicts of Interest

None declared.
